# Cold atmospheric plasma treated medium (PTM) induce antitumor effects in human ovarian cancer cells

**DOI:** 10.1186/s12935-026-04461-6

**Published:** 2026-09-29

**Authors:** Franziska Keßler, Franziska Oelkrug, Ernst Oberlechner, Jürgen Andress, Sara Y. Brucker, André Koch, Daniel A. Carvajal Berrio, Katja Schenke-Layland, Martin Weiss

**Affiliations:** 1 Department of Women’s Health, Eberhard Karls University, 72076 Tübingen, Germany; 2 NMI Natural and Medical Sciences Institute, 72770 Reutlingen, Germany; 3 Institute of Biomedical Engineering, Eberhard Karls University, 72076 Tübingen, Germany

**Keywords:** Ovarian cancer, Ascites, Noninvasive physical plasma, Low thermal argon plasma devitalization, Plasma treated medium, DNA damage, Mitochondrial dysfunction, Anticancer therapy

## Abstract

Plasma treated solutions (PTS) have emerged as a promising anticancer strategy due to their ability to generate reactive oxygen and nitrogen species (RONS) that induce oxidative stress and disrupt multiple cellular pathways. Despite increasing interest in plasma-based cancer therapies, the mechanisms underlying their effects in ovarian cancer remain incompletely understood. This study aimed to investigate the biological effects of plasma treated medium (PTM) in ovarian cancer models, focusing on cell viability, caspase 3/7 activity, DNA damage, cell cycle progression, and mitochondrial metabolism. Methods: Three established ovarian cancer cell lines were treated with PTM to evaluate dose-dependent effects on cell viability using a resazurin assay. Apoptotic signaling was assessed by caspase 3/7 activity assays. DNA damage was analyzed by immunofluorescence staining of γH2AX, while cell cycle alterations were evaluated using propidium iodide staining and flow cytometry. Mitochondrial metabolism was investigated using fluorescence lifetime imaging microscopy together with NADH and FAD autofluorescence imaging. In addition, patient-derived high-grade serous ovarian cancer cells isolated from ascites were analyzed as independent biological replicates to assess biological relevance. Results: PTM induced dose-dependent inhibition of cell viability with IC_50_ values ranging from 1:4 to 1:1.1 PTM dilutions across the tested cell lines. PTM treatment triggered γH2AX-associated DNA damage, caspase 3/7 activity, and dynamic alterations in cell cycle progression. Fluorescence lifetime imaging revealed significant reductions in free NADH and changes in protein-bound NADH and FAD, consistent with mitochondrial dysfunction. These metabolic alterations were accompanied by morphological changes from filamentous mitochondrial networks to punctate structures. Patient-derived ovarian cancer cells showed partial responses, primarily reflected in cell cycle modulation, highlighting both biological relevance and intercellular heterogeneity. Conclusions: PTM exerts multifactorial anticancer effects in ovarian cancer models by inducing DNA damage, caspase 3/7 activity, cell cycle alterations, and mitochondrial dysfunction. These findings provide further insight into the biological effects of plasma-based cancer therapies and support the potential of plasma treated solutions as a novel therapeutic strategy for ovarian cancer.

## Introduction

Ovarian cancer (OvCa) remains one of the most lethal gynecological malignancies worldwide. According to the most recent global estimates, more than 320.000 new cases and over 200.000 deaths from ovarian cancer occurred in 2022, making it one of the leading causes of cancer-related mortality among women with gynecological malignancies [1]. Despite advances in diagnostic procedures and therapeutic strategies, the OvCa prognosis remains poor and exhibits one of the lowest survival rates among gynecological cancers [2]. The extent of surgical cytoreduction remains the single most important prognostic determinant: complete cytoreductive surgery (CRS) expressed by the Completeness of Cytoreduction (CC) Score. CC differentiates between macroscopic tumor clearance (CC0; no visible residual disease) which is strongly associated with improved overall survival, whereas residual macroscopic disease (CC1 (almost complete cytoreduction but visible residual disease) or CC2 (gross residual disease)) significantly worsens outcomes [3–5]. Critically, even after macroscopically complete resection (CC0), occult microscopic tumor deposits may persist at peritoneal surfaces. Novel therapeutic strategies are therefore needed that not only improve outcomes in CC1 or CC2 situations but also enhance the prognostic benefit of CC0 cytoreductive surgery by targeting residual microscopic disease — applicable across both the curative and palliative treatment spectrum of OvCa.

Histologically, nearly 90% of OvCa are classified as epithelial ovarian carcinomas. These tumors can be subdivided into several subtypes including serous (high-grade and low-grade), endometrioid, clear cell and mucinous carcinomas [6]. Among these, high-grade serous carcinoma represents the most common and most aggressive subtype. The etiology of OvCa is multifactorial and includes genetic, hormonal and environmental components. Major risk factors include advanced age, hereditary mutations such as in the BRCA1/2 genes and a family history of breast or ovarian cancer [7, 8]. In contrast, protective factors include multiparity, breastfeeding and long-term use of oral contraceptives, which have been shown to significantly reduce lifetime risk [9].

Standard treatment currently consists of maximal CRS followed by systemic chemotherapy with carboplatin in combination with paclitaxel [10]. The extent of surgical cytoreduction remains a critical prognostic determinant, with complete macroscopic tumor clearance (CC0) being strongly associated with improved survival outcomes. Preliminary data from the phase III TRUST trial (ENGOT ov33/AGOOVAR OP7) suggest a trend toward improved outcomes with primary debulking surgery (PDS) over interval debulking surgery (IDS) following neoadjuvant chemotherapy, particularly in expert centers achieving complete cytoreduction, although the primary endpoint of overall survival was not met [11]. These findings reinforce that CC0 CRS remains the most important surgical goal. Although initial responses to platinum-taxane chemotherapy are high, many patients experience recurrence due to the development of chemoresistance, which significantly limits therapeutic success and contributes to high mortality rates [12]. Furthermore, chemotherapy is frequently associated with systemic toxicity and adverse side effects, highlighting the need for novel therapeutic strategies capable of eliminating residual microscopic disease and thereby enhancing the prognostic benefit of surgical cytoreduction.

Low thermal argon plasma devitalization (LtAPD), a modality of non-invasive physical plasma (NIPP) therapy, has recently emerged as a promising translational approach in oncological research [13–16]. NIPP is a partially ionized gas generated at room temperature that contains a complex mixture of reactive oxygen and nitrogen species (RONS), charged particles, electromagnetic fields, and UV radiation [17]. These components can interact with biological tissues and cells, inducing oxidative stress that selectively affects cancer cells due to their altered redox balance [18]. Increasing evidence demonstrates that LtAPD can exert antitumor effects including inhibition of proliferation, induction of caspase 3/7 activity, DNA damage and disruption of mitochondrial metabolism in various gynecological cancer types such as ovarian cancer, breast cancer and cervical cancer [19–21].

Besides direct plasma application, plasma can also be used indirectly by treating solutions such as cell culture media, thereby generating plasma treated medium (PTM) [22]. Plasma treatment leads to the generation of long-lived reactive species, including hydrogen peroxide, nitrite and nitrate, which can mediate biological effects after application on cells [23]. PTS allows indirect plasma therapy and offers several advantages, including storage, standardized dosing and the possibility of treating locations in the body that are difficult to access with direct treatment.

In the present study, we investigated the in vitro effects of PTM on ovarian cancer cell lines representing different therapy response profiles, including platinum and taxane-resistant phenotypes, as well as on patient-derived primary tumor cells isolated from malignant ascites.

## Materials and methods

### Chemo-resistant and chemo-sensitive human ovarian cancer cells

NIH: OVCAR-3 (ATCC, #HTB161) cells were derived from a high-grade human ovarian adenocarcinoma which is resistant to cisplatin. OVCAR-29 cells originate from an ovarian adenocarcinoma. HEY cells (ATCC, #CRL3252) are derived from a patient with papillary cystadenocarcinoma of the ovary and have been reported to display resistance to taxanes.

### Processing of ascites to obtain primary human ovarian cancer cells

Primary human ovarian cancer cells were isolated from ascites from consenting patients undergoing surgery at the Department of Women’s Health in Tübingen. The Ethical Committee of the Medical Faculty of the Eberhard-Karls-University Tübingen approved the scientific use of the tissue of the Medical Faculty of the Eberhard-Karls-University Tübingen (6492017BO2, approval: 12.01.2018 and 495/2018BO2, approval: 19.10.2018). After surgical collection the ascites was transferred to a falcon and centrifuged at 120 xg for 6 min (Heraeus Multifuge X1R, Thermo Scientific, USA). The supernatant was aspirated and the cell pellet was resuspended in RPMI1640 medium (Merck/Sigma-Aldrich, #R7388) supplemented with 10% heat-inactivated fetal bovine serum (FBS, ThermoFisher Scientific/Gibco™, #16000044) and % Penicillin/Streptomycin (P/S, ThermoFisher Scientific/Gibco™, #15140122) and incubated in a T25 cell culture flask (Greiner BioOne, #690160) at 37 °C and % CO_2_ in an incubator (BBD6220 CO2Inkubator, Thermo Scientific, USA). If the cells reached a confluence ≥ 9% they were partially trypsinized. Therefore, the cells were carefully washed with Dulbecco’s Balanced Salt Solution (DPBS, ThermoFisher Scientific/Gibco™, #14190094), 0.2% Trypsin Ethylenediaminetetraacetic acid (Trypsin/EDTA, Merck/Sigma-Aldrich, # T4049) was added and they were incubated under standard conditions. When the first cells detached, blocking buffer (DPBS + 2% heat-inactivated FBS) was added and the non-adherent cells were aspirated. The adherent cells were washed twice carefully with DPBS and further cultured. This procedure was repeated several times until only ovarian cancer cells remained in the cell culture flask. Primary cultures were expanded only to the extent permitted by their limited proliferative capacity. Ascites-derived primary cultures were prospectively established from eight consecutive patients, covering the histological spectrum of advanced ovarian cancer (high-grade serous, endometrioid, sero-mucinous, and carcinosarcoma). PTM experiments were restricted to the high-grade serous carcinoma (HGSC) cultures, which represent the predominant and clinically most relevant subtype; no pooling of patient-derived cells was performed. The non-HGSC cultures were established and documented using identical procedures but were not included in the functional PTM experiments. Cell-type identity was confirmed by PAX8/Calretinin immunofluorescence staining in two representative HGSC cultures.

### Immunofluorescence staining

To characterize the primary cells obtained from ascites they were analyzed by immunofluorescence staining. 1.75 × 10^4^ cells/cm^2^ of the patient’s samples were seeded in a 12-well Chamber Slide (ibidi, #81201) and incubated for 24 h. Before staining the cells were washed with DPBS three times. For fixation, the cells were incubated with 4% paraformaldehyde (PFA, Merck/Sigma-Aldrich, #158127) in DPBS for 10 min at room temperature (RT) and washed again three times. Afterwards the cells were permeabilized with 0.2% Triton™ X-100 (Merck/SigmaAldrich, #T9284) for 5 min at RT. After washing again, the cells were incubated in 5% blocking buffer (5% bovine serum albumin (BSA, Roth, #8076) in DPBS) for 1 h at RT. Then the cells were incubated with the primary antibodies PAX8 (Proteintech, #103361AP) and Calretinin (Santa Cruz, #sc365956) in a concentration of 1:100 in 0.1% BSA for 1 h. After washing steps, the cells were incubated with the corresponding secondary antibodies Alexa Fluor 594 (anti-rabbit IgG, Thermo Fisher Scientific/Invitrogen, #A11037) and Alexa Fluor 488 (anti-mouse IgG2b, Thermo Fisher Scientific/Invitrogen, #A21141) 1:250 for 1 h at RT protected from light. Also, nuclei were stained with 4’, 6-diamidino-2-phenylindole (DAPI, Thermo Scientific, #62248) diluted 1:500 in DPBS for 20 min at RT. Fluorescence was observed using the Axio Observer Z1 (Carl Zeiss AG, Deutschland).

### Plasma treated solution generation with VIO^®^3/APC3

PTM was generated using an established clinical-grade setup previously described for LtAPD treatment of cervical intraepithelial neoplasia [14–16, 19]. Briefly, 2 ml of RPMI1640 medium without supplements were transferred into a small sterile metal dish. Argon plasma was delivered using the VIO^®^3 electrosurgical generator (Art. No. 10160000, Erbe Elektromedizin GmbH, Tübingen, Germany) in combination with the APC 3 module (Art. No. 10135000) and a 3.2 mm FiAPC probe (Art. No. 20132222), applied in preciseAPC mode, effect 1. The probe was positioned statically at a defined distance of 7 mm from the center of the dish, orthogonal to the medium surface, with a treatment duration of 2 min. Argon served as carrier gas.

### Resazurin assay

OVCAR-3, OVCAR-29, HEY and the primary human ovarian cancer cells were seeded at a density of 3.125 × 10^4^ cells/cm^2^ in a 96-well plate and were cultivated for 24 h at 37 °C and 5% CO_2_ in an incubator. After treatment of the cells with different PTM concentrations for 18 h, Resazurin (Merck/Sigma-Aldrich, #R7017) was used to determine cell viability as previous described [24]. After 1 h the absorbance of the plasma treated samples and the untreated control was measured by using a microplate reader (Spark, Tecan Trading AG, Switzerland).

### CaspaseGlo^®^ 3/7 assay

OVCAR-3, OVCAR-29, HEY as well as isolated primary human ovarian cells (3.125 × 10^4^ cells/cm^2^) were seeded in 96-well plates and were incubated for 24 h at 37 °C and 5% CO_2_. The cells were treated for 4 h with dilutions of PTM generated with the experimental setup. Afterwards the CaspaseGlo^®^ 3/7 Assay (Promega Corporation, #G8090) was performed regarding the instructions of Promega.

### Analysis of double strand breaks

OVCAR-3, OVCAR-29, HEY and isolated primary human ovarian cells were seeded at a density of 1.56 × 10^4^ cells/cm^2^ in a 6-well plate and were incubated for 24 h at 37 °C and 5% CO_2_. Cells were treated with the cell-type-specific IC_50_ PTM dilutions generated with the previously described experimental setup and incubated for 18 h. Cells were trypsinized and resuspended in 500 µl ice cold DPBS. Afterwards 4 ml ice cold 1% PFA was added and cells were incubated on ice for 15 min. After washing and centrifugation cells were transferred dropwise into 2 ml 70% EtOH and were incubated at – 20 °C overnight. The next day cells were washed and incubated in 2 ml 1% BSA and 0.2% Triton™ X-100 diluted in DPBS for 5 min at RT. Afterwards cells were resuspended in γH2AX staining solution (1 µg/ml γH2AX antibody (Merck/Sigma-Aldrich, #16202A) in BSA-Triton buffer). Staining was performed for 1 h at RT protected from light. Cells were analyzed in a BD LSRFortessa™ Cell Analyzer flow cytometer (BD Bioscience, Germany).

### Cell cycle analysis

OVCAR-3, OVCAR-29, HEY and isolated primary human ovarian cells were seeded at a density of 1.56 × 10^4^ cells/cm^2^ in a 6-well plate and were incubated for 24 h at 37 °C and 5% CO_2_. Cells were treated with the cell-type-specific IC_50_ PTM dilutions generated with the previously described experimental setup and incubated for 1 h. After trypsinization and centrifugation cells were resuspended in 500 µl ice cold DPBS. Afterwards 4 ml ice cold 1% PFA was added and cells were incubated on ice for 15 min. After washing and centrifugation cells were transferred dropwise into 2 ml 70% EtOH and were incubated at – 20 °C overnight. The next day cells were washed and incubated in 2 ml 1% BSA and 0.2% Triton™ X-100 diluted in DPBS for 5 min at RT. Afterwards cells were resuspended in a 3 µM propidium iodide staining solution (PI (Thermo Fisher/Invitrogen, #P1304MP) in BSA-Triton buffer). Staining was performed for 30 min at RT protected from light. Cells were analyzed in a BD LSRFortessa™ Cell Analyzer flow cytometer (BD Bioscience, Germany).

### Fluorescence lifetime imaging microscopy (FLIM)

1.92 × 10⁴ cells/cm² of OVCAR-29, HEY and isolated primary human ovarian cancer cells were seeded and incubated for 24 h prior to PTM treatment at cell-type-specific IC_50_ PTM dilutions generated with the experimental setup.

### NADH and FAD autofluorescence imaging

To assess mitochondrial metabolic activity, unlabeled cells were analyzed by FLIM based on the intrinsic autofluorescence of the mitochondrial coenzymes NADH and FAD. Images were acquired using a laser scanning microscope with a laser power of 0.2% and a 63× C-Plan Apochromat/1.4 oil immersion objective.

### Mitochondrial membrane potential (ΔΨm)

To evaluate mitochondrial membrane potential, cells were stained with tetramethylrhodamine (TMRM, Thermo Fisher Scientific/Invitrogen™, #T668) following PTM treatment, according to the manufacturer’s instructions for 30 min. TMRM accumulates in mitochondria in a membrane potential-dependent manner and was imaged by FLIM using the same laser scanning microscope setup as described above.

### Statistical analysis

All experiments were repeated with at least three independent biological replicates. Each biological replicate represents the mean of three technical replicates. Date are represented as mean ± Standard Deviation (SD). Microsoft Excel, Microsoft Power Point and GraphPad Prism version 9.4.1 were used for the statistical analyses. To compare two groups an unpaired Student’s t test was performed. For comparisons between more than two groups a one-way or two-way ANOVA test was performed. Statistical significance was defined as follows: p-values > 0.05 indicate no significance (ns), p-values < 0.05 indicate significance (*).

## Results

### Isolation and detection of serous ovarian cancer cells from ascites

**Fig. 1:**
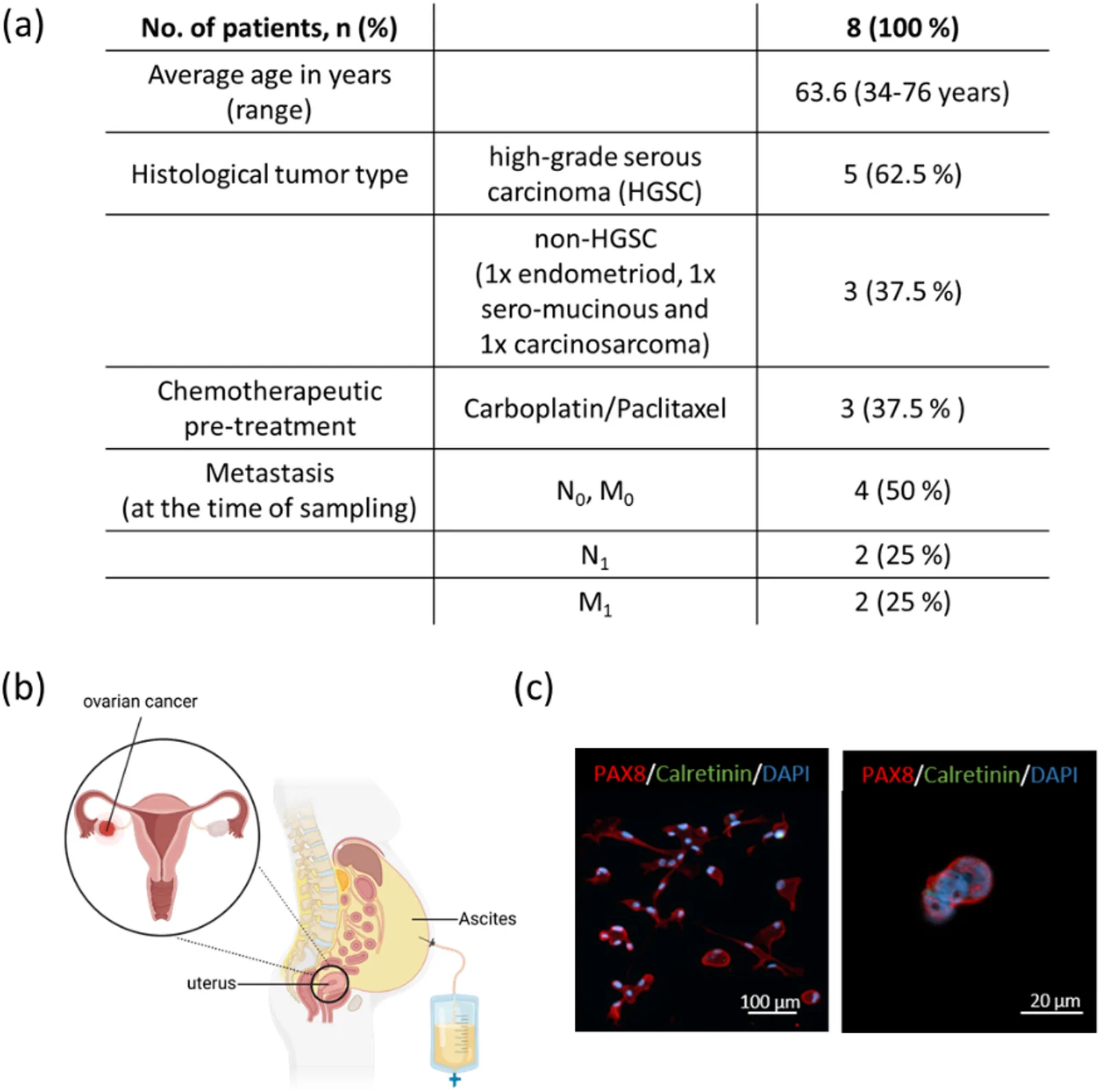
Ascites-derived ovarian cancer cells. (**a**) Classification of diagnosed ovarian cancer patients with regard to age, histological tumor type, chemotherapeutic pretreatment and metastasis. (**b**) Scheme of ascites sampling in patients with ovarian cancer. (**c**) Representative immunofluorescence staining images of the isolated cells from ascites with the ovarian carcinoma marker PAX8 (red) and the mesothelial cell marker calretinin (green). Cell nuclei are stained using DAPI (blue). Scale bars are indicated in the image

Eight samples were collected from patients suffering from ascites as a result of diagnosed ovarian cancer (Fig. 1(a)). The patients were on average 63.6 years old. Five patients (62.5%) had high-grade serous carcinoma (HGSC), whereas the remaining three patients (37.5%) presented with non-HGSC histologies (one endometrioid, one sero-mucinous, and one carcinosarcoma). Functional PTM experiments were subsequently restricted to the HGSC cultures (see Materials and Methods). Three (37.5%) of the eight patients had undergone chemotherapeutic pretreatment with paclitaxel in combination with carboplatin, while the remaining five patients had not received any prior chemotherapy. Lymph node involvement near the tumor was detected in two patients (25%) and distant metastases were found in two patients (25%) at the time of sampling. The remaining four patients (50%) were free of distant metastases (Table in Fig. 1(a)).

After collection, the samples were centrifuged, supernatant was aspirated and cells were resuspended in cell culture medium and cultured under standard conditions. When the cells were almost confluent, they were partially trypsinized and detached cells were washed away. This procedure was repeated several times until only ovarian cancer cells remained in the culture (Fig. 1(b)).

Subsequently, a cell-type-specific immunofluorescence staining was performed (Fig. 1(c)). Positive PAX8 staining in combination with a negative Calretinin staining serves as a sensitive indicator for carcinomas with ovarian origin. Calretinin alone is a reliable marker for mesothelial cells [26]. Approximately 90% of the isolated cells were PAX8 positive and Calretinin negative (*n* = 2). PAX8-negative and Calretinin-positive cells were not detected in the samples. A minor fraction (~ 5%) co-expressed both markers. These results confirmed, that the majority of patient-derived cells isolated from ascites were ovarian cancer cells, which could be further used to assess the effects of PTM on cell viability, cell cycle progression and cell metabolism.

### Antiproliferation effects of PTM on ovarian cancer cell lines

**Fig. 2:**
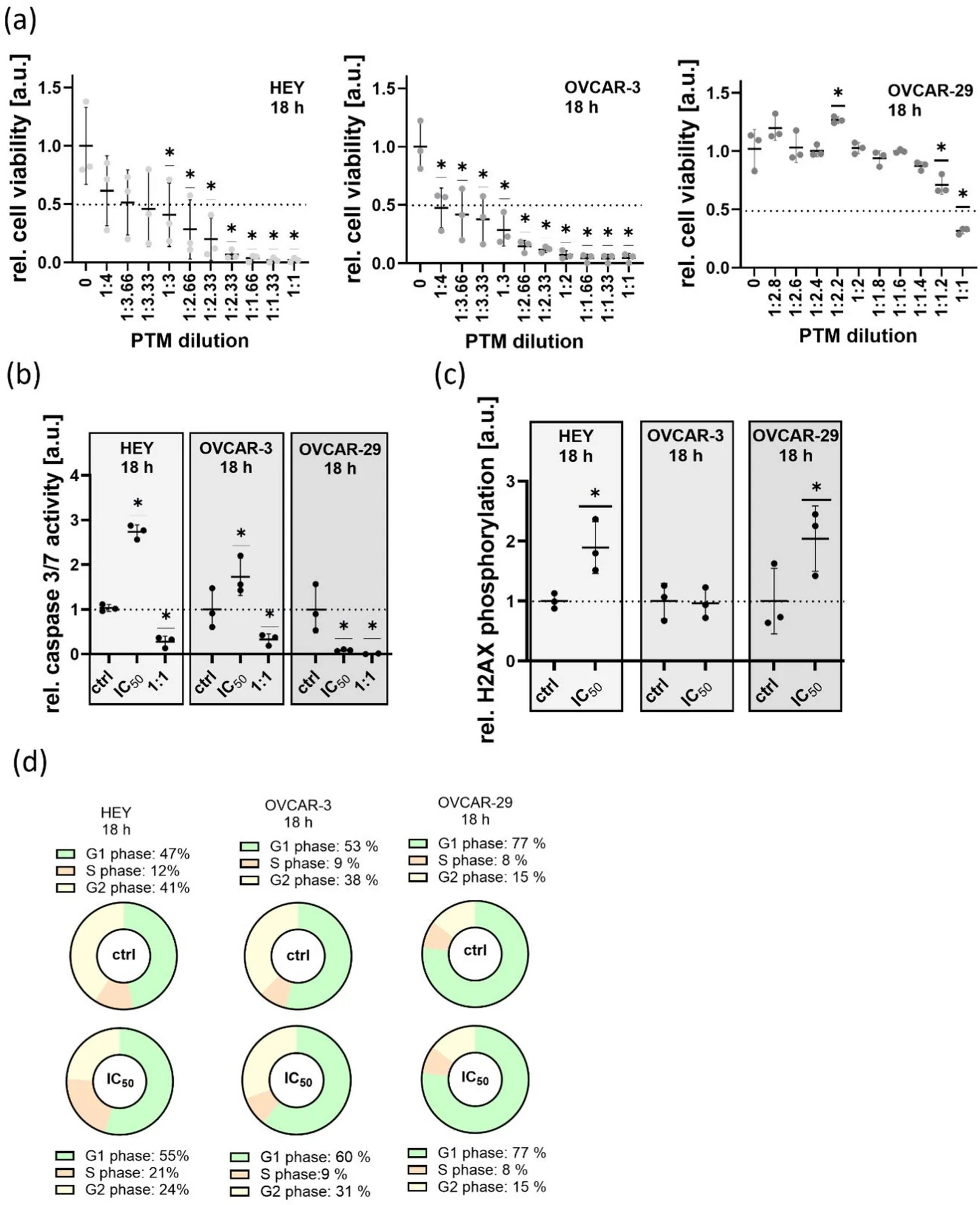
PTM treatment affects cell viability, caspase 3/7 activity and causes DNA damage in ovarian cancer cell lines. (**a)** Relative cell viability of HEY, OVCAR-3 and OVCAR-29 after 18 h of PTM treatment. IC_50_ for the three cell lines were determined: HEY (1:3.6 PTM dilution), OVCAR-3 (1:4 PTM dilution) and OVCAR-29 (1:1.1 PTM dilution). (**b**) Relative caspase 3/7 activity of cells after treatment with IC_50_ and 1:1 PTM dilution for 18 h. (**c**) Relative H2AX phosphorylation after treatment with cell-type-specific IC_50_ for 18 h. (**d**) Cell cycle arrest after IC_50_ PTM treatment for 18 h. Results are shown as mean ± SD, and individual values for each biological replicate are indicated. Significance (*): *p* < 0.05 as determined by ANOVA test (ordinary one-way or two-way)

Since chemotherapies often have severe side effects and relapses occur particularly in patients in advanced stages, therapeutic approaches are needed that are not leading to the development of resistance. To investigate the antiproliferative efficacy of PTM on ovarian cancer cells, established cell lines (OVCAR-29, OVCAR-3, HEY) were treated for 18 h with serial dilutions of plasma treated medium (PTM). Cell viability was significantly reduced after treatment of ovarian cancer cells (HEY: 1:3 PTM dilution, OVCAR-3: 1:3 PTM dilution, OVCAR-29: 1:1.2 PTM dilution). Cell-type-specific IC_50_ values were as follows for the cell lines: 1:3.6 PTM dilution for HEY and 1:4 PTM dilution for OVCAR-3 and 1:1.1 PTM dilution for OVCAR-29 (Fig. 2(a)). Cell-type-specific IC_50_ and undiluted PTM (1:1) were used for further investigations. To investigate whether PTM activates apoptotic signaling pathways, caspases 3/7 activity was measured after 18 h of treatment. HEY cells exhibited significant increases in effector caspase activity. OVCAR-3 cells showed an upward trend that did not reach significance, while OVCAR-29 cells demonstrated reduced overall activity, reflecting extensive cell loss at high PTM concentrations (Fig. 2(b)). To investigate whether DNA damage contributes to the activation of apoptotic pathways in ovarian cancer cells, H2AX phosphorylation was examined 18 h after PTM treatment. For OVCAR-29 and HEY cells, an increase in H2AX phosphorylation could be detected 18 h after PTM treatment. In OVCAR-3 cells, H2AX phosphorylation could not be demonstrated under these conditions (Fig. 2(c)). To examine the effect of DNA damage after PTM treatment on the cell cycle, the distribution of treated cells in the G1-, S- and G2 phases was determined. After PTM treatment, a change in the number of cells in the G1 phase was detected for HEY and OVCAR-3 cells. After 18 h of treatment, the percentage of cells in the G1 phase increased slightly but not statistically significant after treatment (for HEY cells by % and for OVCAR-3 cells by %). Inversely proportional to this, the percentage of cells in the G2 phase decreased (HEY: 1%, OVCAR-3: %). For HEY cells the portion of cells in S phase increased slightly by %. In OVCAR-29 cells there was no effect observed (Fig. 2(d)).

### Antiproliferation effects of PTM on primary ovarian cancer cells isolated from ascites

**Fig. 3:**
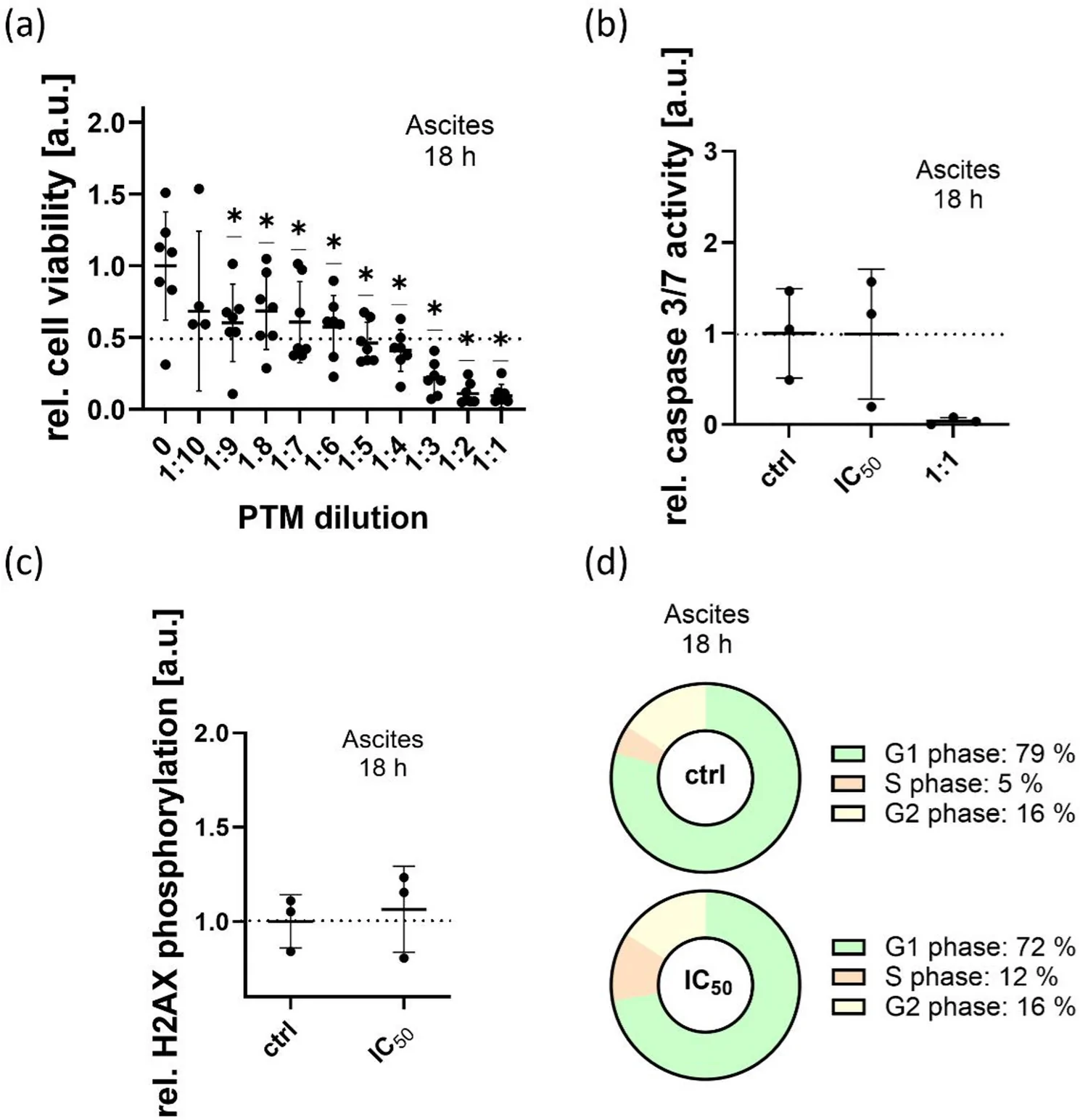
PTM treatment affects cell viability, caspase 3/7 activity and causes DNA damage in ascites-derived ovarian cancer cells. (**a**) Relative cell viability of primary ovarian cancer cells after 18 h of PTM treatment. IC_50_ was determined at a PTM dilution of 1:5. (**b**) Relative caspase 3/7 activity of cells after treatment with IC_50_ for 18 h. (**c**) Relative H2AX phosphorylation after treatment with IC_50_ for 18 h. (**d**) Changes in cell cycle after IC_50_ PTM treatment for 18 h. Results are shown as mean ± SD, and individual values for each biological replicate are indicated. Significance (*): *p* < 0.05 as determined by ordinary one-way ANOVA test

For a potential future clinical application, it was examined whether the plasma effects can also be observed in HGSC primary cells isolated from ascites of patients suffering from ovarian cancer. Again, after 18 h of treatment with PTM diluted 1:9 a significant reduction of cell viability of approximately 40% was observed. IC_50_ was determined at a PTM dilution of 1:5 PTM (Fig. 3(a)). No increase in caspase 3/7 activity was detected after PTM treatment (Fig. 3(b)). Furthermore, no significant increase in H2AX phosphorylation was observed compared to untreated cells (Fig. 3(c)). Cell cycle distribution showed a shift from G1 phase to S Phase after 1:5 PTM dilution treatment. Cells in G1 phase decreased from 79% in untreated control cells to 72% in treated cells and cells in S phase increased from 5% to 12%, while number of cells in G2 phase remained largely unchanged (Fig. 3(d)).

### PTM leads to a mitochondrial dysfunction in ovarian cancer cells

**Fig. 4:**
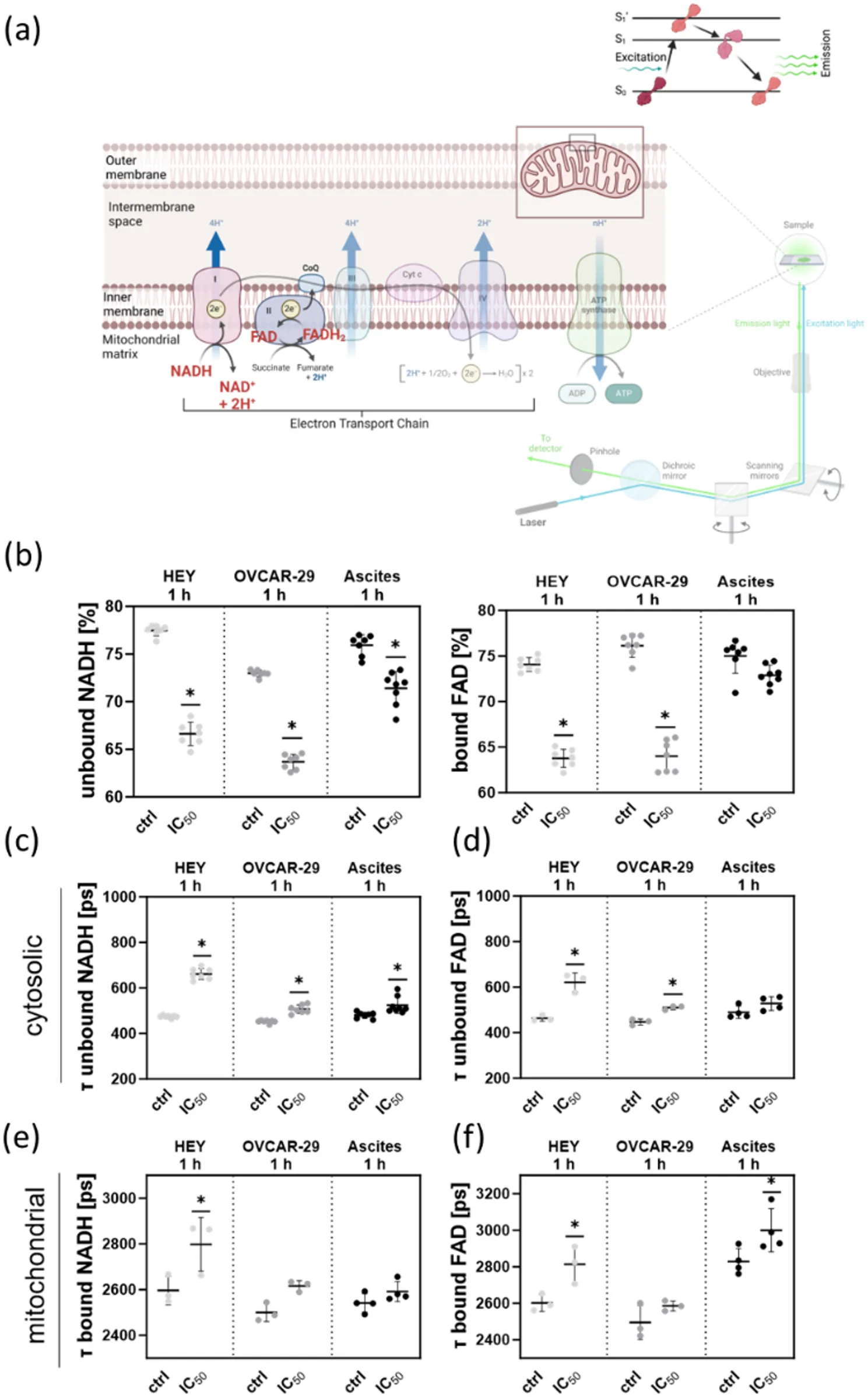
Effects of PTM treatment on redox cell metabolism in ovarian cancer cells. (**a**) Schema of the respiratory chain in the cell, which is examined with the aid of fluorescence lifetime microscopy (FLIM). Light of a certain wavelength is used to excite molecules into a higher energy state. The time that the molecule needs to return to its original energy state is referred to as the fluorescence lifetime τ [ps]. Since the electron carriers NADH and FAD of the respiratory chain are autofluorescent, they are suitable for analyzing metabolism. (**b**) Percentage of free NADH and bound FAD in the total occurrence in HEY, OVCAR-29 and ascites cells. Fluorescence lifetime τ [ps] of free (**c**) NADH and (**d**) FAD in the cytosol and bound (**e**) NADH and (**f**) FAD in the mitochondria of HEY, OVCAR-29 and ascites cells after PTM treatment using the cell-type-specific IC_50_ after an incubation time of 1 h. Cells were analyzed by laser microscopy after PTM treatment. Results are shown as mean ± SD, and individual values for each biological replicate are indicated. Significance (*): *p* < 0.05 as determined by two-way ANOVA test

To investigate the effects of PTM on cellular metabolism, the mitochondrial coenzymes NADH and FAD were analyzed by fluorescence lifetime imaging microscopy (FLIM). Both coenzymes are integral components of the respiratory chain, serving as electron carriers during oxidative phosphorylation. Their intrinsic autofluorescence properties allow non-invasive assessment of metabolic activity: the fluorescence lifetime τ [ps] reflects the molecular binding state and metabolic context of each coenzyme, enabling discrimination between free (cytosolic) and protein-bound (mitochondrial) fractions (Fig. 4(a)). HEY cells, OVCAR-29 cells and primary ovarian cancer cells isolated from ascites were treated with PTM at cell-typespecific IC_50_ PTMdilution for 1 h prior to analysis.

Regarding the distribution of coenzyme fractions, PTM treatment induced a significant reduction of approximately 10% in the proportion of free NADH in HEY and OVCAR-29 cells and 3% in primary ovarian cancer cells (Fig. 4(b), left). Similarly, the proportion of protein-bound FAD was significantly reduced by approximately 1% in HEY and 1% in OVCAR-29 cells, while no significant effect was observed in ascites-derived cells (Fig. 4(b), right).

With respect to fluorescence lifetimes, the lifetime of free, cytosolic NADH was significantly prolonged following PTM treatment in all three cell types (Fig. 4(c)). For protein-bound, predominantly mitochondrial NADH, a significant increase was observed in HEY cells, a non-significant trend was noted in OVCAR-29 cells and no relevant change was detected in ascites-derived cells (Fig. 4(e)). A comparable pattern was observed for FAD: the fluorescence lifetime of free, cytosolic FAD was significantly prolonged in HEY and OVCAR-29 cells but remained unchanged in ascites-derived cells (Fig. 4(d)), whereas the lifetime of protein-bound, predominantly mitochondrial FAD was significantly increased in HEY cells and ascites-derived cells, with no significant difference detected in OVCAR-29 cells (Fig. 4(f)).

Collectively, these findings indicate an imbalance between electron carrier formation and their oxidation in the respiratory chain, consistent with impaired mitochondrial oxidative phosphorylation following PTM treatment. The attenuated response observed in primary ascites-derived cells suggests enhanced metabolic resilience compared to established cell lines.

### PTM induces changes in the mitochondrial transmembrane potential (ΔΨm)

To assess whether the observed imbalance of the electron carriers following PTM treatment resulted in an altered mitochondrial transmembrane potential, the cells were stained with TMRM after PTM treatment and its fluorescence lifetime was determined. TMRM accumulates in the mitochondria in a membrane potential dependent manner: a high membrane potential drives stronger dye accumulation, which shortens the fluorescence lifetime due to aggregation effects. Accordingly, a prolonged TMRM fluorescence lifetime is indicative of mitochondrial membrane depolarization.

**Fig. 5:**
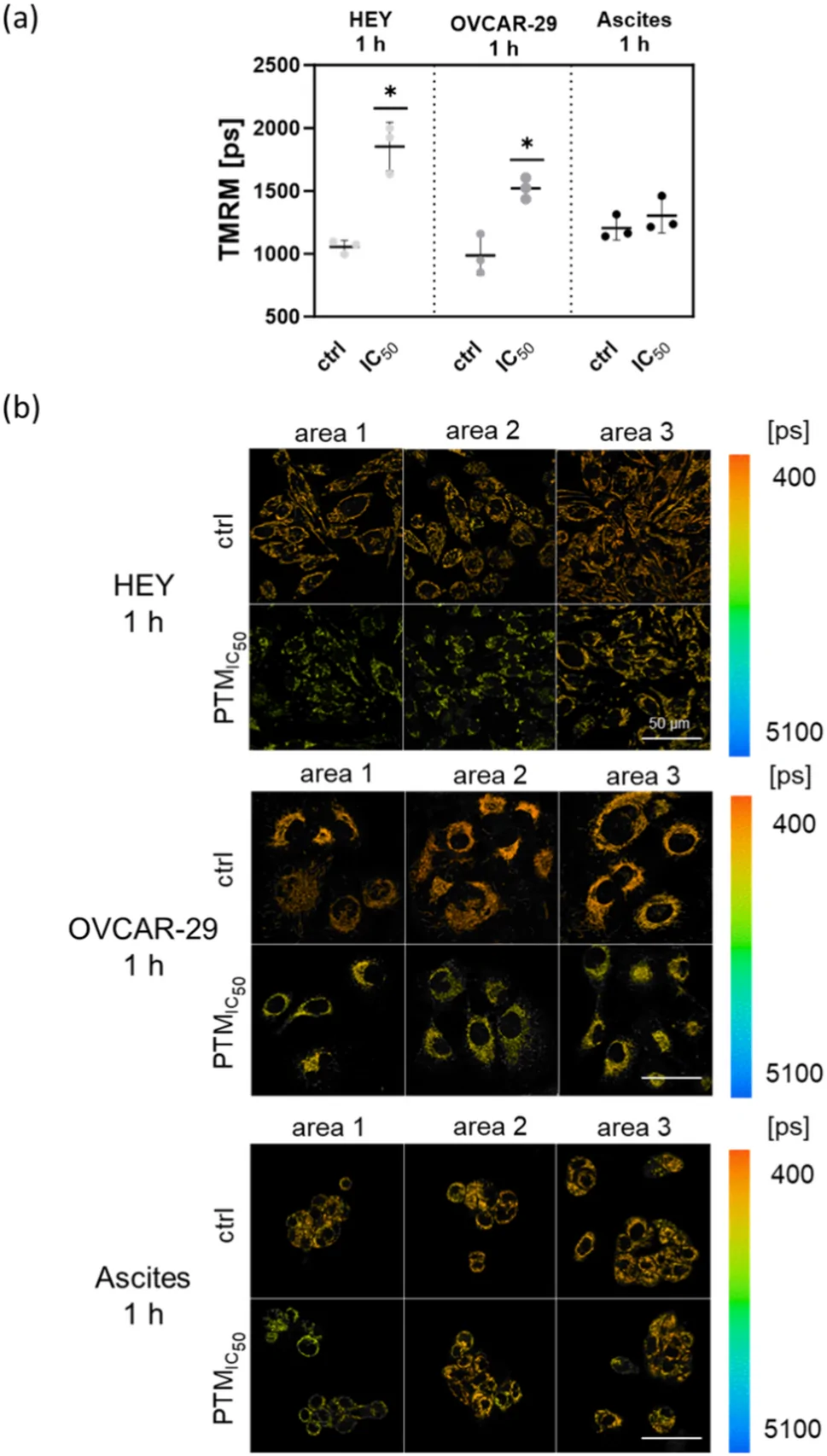
Effects of PTM treatment on the mitochondrial transmembrane potential. (**a**) Tetramethylrhodamine (TMRM) fluorescence lifetime τ [ps] in HEY, OVCAR-29 and ascites cells and (**b**) representative laser microscopy of HEY, OVCAR-29 and ascites cells after PTM treatment for 1 h using the cell-type-specific IC_50_ and untreated cells. Cells were stained with TMRM after PTM treatment. Results are shown as mean ± SD, and individual values for each biological replicate are indicated. Significance (*): *p* < 0.05 as determined by two-way ANOVA test. Scale bars represent 50 μm

Following PTM treatment, the fluorescence lifetime of TMRM was significantly increased in HEY and OVCAR-29 cells, indicating membrane depolarization, while no significant difference was detected in primary ascites-derived cells compared to untreated controls (Fig. 5(a)).

Beyond the lifetime measurements, pseudocolor-coded FLIM images additionally allowed assessment of mitochondrial morphology (Fig. 5(b)). In HEY cells, a clear shift in signal color from orange in untreated controls to yellow-green after PTM treatment reflected the prolonged fluorescence lifetime. Concurrently, the physiological filamentous, thread-like mitochondrial network of untreated HEY cells was replaced by punctate, partly vesicular structures following treatment. A comparable morphological transition from filamentous to punctiform mitochondrial architecture was observed in OVCAR-29 cells. In contrast, ascites- derived cells showed neither relevant changes in color coding nor discernible alterations in mitochondrial morphology after PTM treatment.

## Discussion

Ovarian cancer remains one of the most lethal gynecologic malignancies due to late diagnosis, peritoneal dissemination, and frequent chemoresistance [6, 25, 26]. Novel approaches that selectively target tumor cells while minimizing systemic toxicity are urgently needed. PTM has emerged as a promising strategy, generating RONS that induce oxidative stress, DNA damage, and disruption of cellular metabolism in cancer cells [27, 28]. In this study, we examined the effects of PTM on both established ovarian cancer cell lines and primary tumor cells derived from patients’ ascites.

Treatment of established ovarian cancer cell lines revealed marked differences in PTM sensitivity, with IC_50_ values ranging from 1:4 to 1:1.1 PTM dilution. HEY and OVCAR-3 cells were highly susceptible, whereas OVCAR-29 cells required substantially higher PTM concentrations to achieve comparable cytotoxicity. These differences likely reflect intrinsic variations in antioxidant defenses, redox buffering, and DNA repair capacity. Interestingly, the chemo-resistant OVCAR-3 cells exhibited substantially higher PTM sensitivity than the non-chemo-resistant OVCAR-29 cells. This observation suggests that resistance to conventional chemotherapy does not necessarily confer resistance to PTM-derived reactive oxygen and nitrogen species [29]. Thus, susceptibility to PTM appears to be determined by factors beyond the chemo-resistant phenotype itself. Differences in antioxidant capacity, redox buffering, metabolic dependencies and DNA repair pathways may contribute to these differential responses. These findings indicate that PTM may target vulnerabilities distinct from those affects by conventional chemotherapeutic agents and warrant further mechanistic investigation [29, 30]. In sensitive cell lines, RONS accumulation appears to rapidly exceed the cellular antioxidant threshold, leading to oxidative DNA damage, mitochondrial stress and activation of apoptotic signaling pathways, as evidenced by caspase 3/7 activation and H2AX phosphorylation. In OVCAR-3 cells, caspase activation showed only an upward trend without reaching significance, and H2AX phosphorylation was not detectable under the applied conditions, suggesting that DNA damage-independent mechanisms may contribute to PTM-induced cytotoxicity in this cell line, or that the applied IC_50_ PTM dilution was insufficient to trigger detectable genotoxic signaling within the observed time frame. Primary ascites-derived cells exhibited minimal caspase activation and H2AX phosphorylation despite reduced viability, suggesting that these cells employ adaptive survival mechanisms — potentially including robust DNA repair capacity, stem cell-like characteristics, and metabolic plasticity — that allow them to withstand oxidative stress and circumvent immediate apoptosis [19].

Cell cycle analysis further illustrated these differential responses. Established lines generally accumulated in G1 with a concomitant decrease in G2 phase, indicative of checkpoint activation in response to oxidative DNA damage, while primary ascites-derived cells shifted from G1 to S phase. This may reflect slowed S phase progression or alternative cell cycle adaptation, consistent with stress-resilient or chemo-resistant tumor populations observed in advanced OvCa [31]. Together, these findings highlight that the efficacy of PTM depends not only on the applied dose but also on intrinsic cellular heterogeneity, with primary cells showing transient survival strategies absent in immortalized lines.

PTM also induced significant metabolic perturbations. In HEY and OVCAR-29 cells, fluorescence lifetime measurements revealed decreases in free NADH, increases in protein-bound NADH lifetimes, and alterations in FAD lifetimes, accompanied by mitochondrial membrane depolarization and fragmentation. These observations indicate that PTM disrupts oxidative phosphorylation and mitochondrial bioenergetics, likely contributing to the activation of apoptosis or necrosis [27, 28, 32]. In primary ascites-derived cells, mitochondrial and metabolic effects were less pronounced, suggesting enhanced redox resilience and metabolic adaptability, which may reflect the survival strategies of tumor cells conditioned to the oxidative peritoneal microenvironment. Although the observed changes in caspase 3/7 activity, H2AX phosphorylation, mitochondrial membrane potential, and metabolic parameters suggest the involvement of apoptosis-associated signaling and mitochondrial dysfunction, the present study was designed primarily to characterize the phenotypic response to PTM. Consequently, mechanistic intervention experiments, such as ROS scavenging, caspase inhibition, or modulation of mitochondrial function, were not performed. In addition, the present study relied mainly on functional assays and imaging-based analyses and did not include validation approaches, such as immunoblotting of apoptosis- or mitochondrial-associated proteins. Therefore, the observed alterations in caspase activity and mitochondrial metabolism could not be confirmed by independent complementary methods. Additionally, causal relationships between oxidative stress, mitochondrial impairment, and cell death-associated signaling cannot be conclusively established. Future studies employing complementary methodologies, including Western blot analysis, as well as targeted gain- and loss-of-function approaches, will be required to further substantiate the molecular mechanisms underlying PTM-induced cytotoxicity.

Across all models, PTM generates RONS that challenge cellular antioxidant defenses, leading to oxidative stress. Sensitive cells accumulate DNA damage, undergo cell cycle arrest, experience mitochondrial dysfunction, and ultimately activate apoptosis-related signaling pathways. More resilient cells, such as primary ascites-derived tumor cells, may initially survive through adaptive mechanisms, yet show slowed proliferation, induced metabolic stress, and sustained vulnerability to higher PTM concentrations. Conceptually, PTM functions as a multimodal stressor that may exploit vulnerabilities associated with malignant cell metabolism and redox homeostasis.

The present study has several limitations. All experiments were conducted in vitro, and findings require validation in appropriate in vivo models. Primary ascites-derived cultures were prospectively established from a cohort of eight patients spanning several histological subtypes. The functional PTM analyses were restricted to the HGSC subset. Consequently, the present findings apply primarily to HGSC, and their generalizability to non-HGSC subtypes and to the broader spectrum of tumor heterogeneity remains to be established. A detailed physicochemical characterization of the PTM batches used in the present study was not performed; however, given the use of an identical VIO^®^3/APC3 setup, the RONS composition can be reasonably inferred from previous characterization work by our group [19]. Additionally, no direct comparison with standard chemotherapeutic agents was performed. Furthermore, non-malignant ovarian epithelial cells or primary cultures derived from healthy tissue were not included in the present study. Therefore, no conclusions regarding the selectivity of PTM for malignant versus non-malignant ovarian cells, or its potential toxicity to healthy tissue can be drawn in this study. Direct comparison of malignant and non-malignant ovarian cells will be essential to define the therapeutic window and further evaluate the translational potential of PTM for ovarian cancer treatment.

Despite these limitations, the findings have valuable clinical implications. The extent of surgical cytoreduction remains the single most important prognostic factor in ovarian cancer, with CC0 CRS being strongly associated with improved survival. However, even after macroscopically complete resection, occult microscopic tumor deposits may persist at peritoneal surfaces, and CC1 and CC2 remain common in advanced stage disease (FIGO stage IIIIV). In this context, PTM may represent a complementary therapeutic strategy: improving outcomes in CC1 and CC2 through targeted elimination of residual tumor cells, as well as enhancing the prognostic benefit of CC0 CRS-cytoreductive surgery by addressing occult microscopic disease. The observed variability among cell types underscores the importance of personalized approaches and mechanistic understanding of redox, metabolic, and cell cycle vulnerabilities. Overall, PTM induces oxidative and metabolic stress responses associated with reduced proliferation in ovarian cancer models.

## Conclusions

PTM treatment induces significant antiproliferative effects in ovarian cancer cells through a combination of oxidative stress, mitochondrial dysfunction, and cell cycle disruption. While established chemo-resistant cell lines exhibited increased caspase 3/7 activity consistent with activation of apoptotic pathways, primary ascites-derived cells displayed adaptive survival mechanisms reflecting the inherent heterogeneity of patient tumors. These differential responses underscore the importance of personalized therapeutic strategies targeting redox and metabolic vulnerabilities. In the clinical context of ovarian cancer, where complete cytoreductive surgery remains the most critical prognostic determinant, PTM holds promise as a complementary approach to address residual microscopic and macroscopic disease in both settings CC0 as well as CC1 and CC2. Future studies should validate these findings in vivo and explore the combination of PTM with standard chemotherapeutic regimens to fully realize its therapeutic potential. 

## Data Availability

Data Availability Statement: Dataset available on request from the authors.

## Abbreviations

CCx: Completeness of Cytoreduction
CRS: Complete cytoreductive surgery
FLIM: Fluorescent lifetime imaging microscopy
HGSC: High-grade serous carcinoma
LtAPD: Low thermal argon plasma devitalization
NIPP: Noninvasive physical plasma
OvCa: Ovarian Cancer
PTM: Plasma treated medium
PTS: Plasma treated solutions
RONS: Reactive oxygen and nitrogen species
TMRM: Tetramethylrhodamine
